# Multiple anthropometric characteristics and Brugada syndrome: A Mendelian randomization study

**DOI:** 10.1097/MD.0000000000044990

**Published:** 2025-10-10

**Authors:** Sina Liu, Juju Shang, Hongxu Liu, Wenlong Xing, Wei Liu, Shenglei Qiu, Xiaolei Lai, Mingxuan Li, Siqing Wang

**Affiliations:** a Beijing University of Chinese Medicine, Beijing, China; b Department of Cardiovascular, Beijing Hospital of Traditional Chinese Medicine, Capital Medical University, Beijing, China.

**Keywords:** anthropometry, Brugada syndrome, causality, Mendelian randomization, risk factor

## Abstract

Brugada syndrome (BrS) has been associated with lower obesity rates and reduced body mass index (BMI) in observational studies. This Mendelian randomization (MR) study investigates causal effects of anthropometric traits on the risk of BrS. Two-sample MR analyses were conducted using publicly available genome-wide association study summary statistics, and both discovery and replication datasets were incorporated. The inverse-variance weighted method was the primary analytical approach, supported by MR-Egger and weighted median methods. Sensitivity analyses (including Cochran Q test, MR-Egger intercept test, MR-PRESSO global test, and leave-one-out analysis) were performed to assess the robustness of the results. Genetically predicted comparative body size at age 10 showed significant inverse associations with BrS risk (discovery: odds ratio [OR] = 0.40, 95% confidence interval [CI]: 0.26–0.61, *P* = 2.07 × 10^−5^; replication: OR = 0.47, 95% CI: 0.31–0.71, *P* = 3.68 × 10^−4^). Similarly, adult obesity was inversely associated with BrS in the discovery set (OR = 0.78, 95% CI: 0.69–0.89, *P* = 1.42 × 10^−4^). Genetically predicted childhood obesity, whole-body fat-free mass, BMI, waist-to-hip ratio, and basal metabolic rate also demonstrated potential protective effects (discovery: all *P* < .05). Reverse MR analysis revealed positive associations between genetically predicted BrS and both waist and hip circumference (*P* < .005). This study suggests that genetically higher childhood/adult obesity and increased fat-free mass may reduce the risk of BrS. Maintaining a BMI in the upper-normal range and enhancing fat-free mass through resistance training may represent a viable strategy to mitigate BrS risk.

## 1. Introduction

Brugada syndrome (BrS) is a cardiac channelopathy predominantly inherited in an autosomal dominant manner and was first described in 1992.^[1]^ Clinically, BrS accounts for approximately 20% of sudden cardiac deaths in individuals without structural heart disease.^[2,3]^ It typically manifests in adulthood, with a mean age of sudden death at 41 ± 15 years,^[4]^ and is observed 8 to 10 times more frequently in males than in females. Epidemiological data indicate that the prevalence of BrS is higher in Asians (0.94%) compared to Europeans (0.02%). Diagnosis requires characteristic electrocardiographic features, with type 1 BrS presenting as a coved-type ST-segment elevation in the right precordial leads. Various factors can trigger BrS manifestations, including antiarrhythmic drugs, sodium channel blockers, psychotropic agents, anesthetics, cocaine, vagotonic states, metabolic disturbances, electrolyte imbalances, excessive alcohol intake, overeating, and fever. Risk stratification for adverse outcomes (such as ventricular tachycardia, ventricular fibrillation, and cardiac arrest) includes predictors such as a history of syncope, aborted sudden cardiac death, a spontaneous type 1 BrS ECG pattern, sinus node dysfunction, a family history of sudden cardiac death in first-degree relatives, and inducible ventricular arrhythmias. Additionally, patients carrying pathogenic mutations in *SCN5A* or other genes associated with loss-of-function variants also show an increased risk of ventricular arrhythmia.^[2,3]^

Epidemiological studies suggest that BrS patients generally have lower body mass index (BMI), reduced body fat percentage, and lower obesity prevalence compared to non-BrS populations.^[5–7]^ However, the low prevalence of BrS results in limited sample sizes, and observational designs remain vulnerable to confounding, reverse causality, and measurement bias, hindering definitive conclusions regarding causality.

Mendelian randomization (MR) analysis, which leverages genetic variants as instrumental variables to estimate the causal effects of modifiable exposures on disease outcomes,^[8]^ can minimize the confounding, reverse causation, and measurement errors inherent in observational studies.^[9,10]^ Previous MR studies have established causal associations between anthropometric traits and cardiovascular disease risk. For example, birth weight,^[11]^ childhood obesity, childhood BMI,^[12]^ adult BMI, waist-to-hip ratio (WHR),^[13]^ fat mass index, fat-free mass index,^[14]^ whole-body water mass (WBWM),^[15]^ and basal metabolic rate (BMR)^[16]^ have all been linked to various cardiovascular outcomes. However, no MR study to date has specifically examined the causal relationships between these anthropometric traits and BrS.

Therefore, this MR study systematically evaluated the causal associations between genetically predicted obesity-related anthropometric characteristics and the risk of BrS, aiming to inform clinical management and public health strategies.

## 2. Materials and methods

### 2.1. Study design

**Figure 1.**
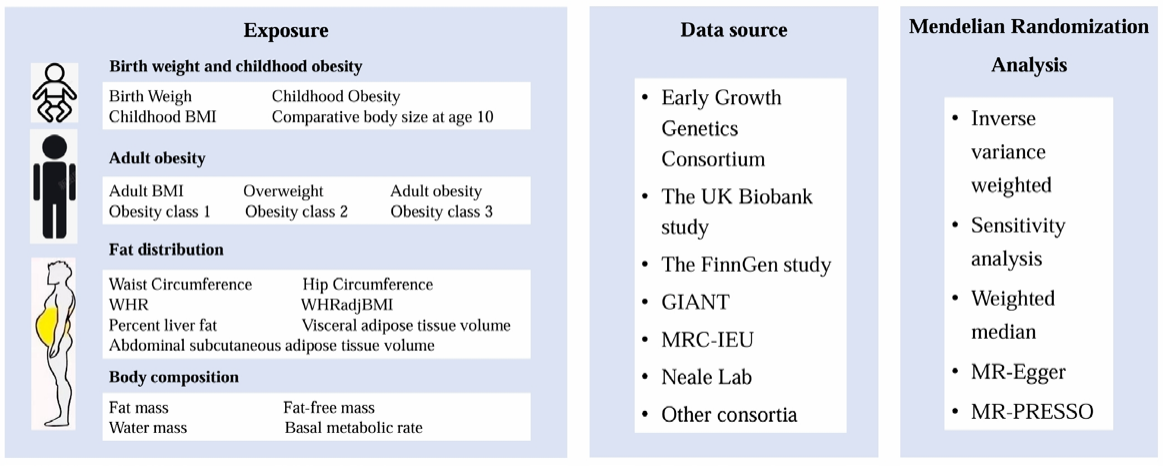
Flowchart of Mendelian randomization.

**Figure 2.**
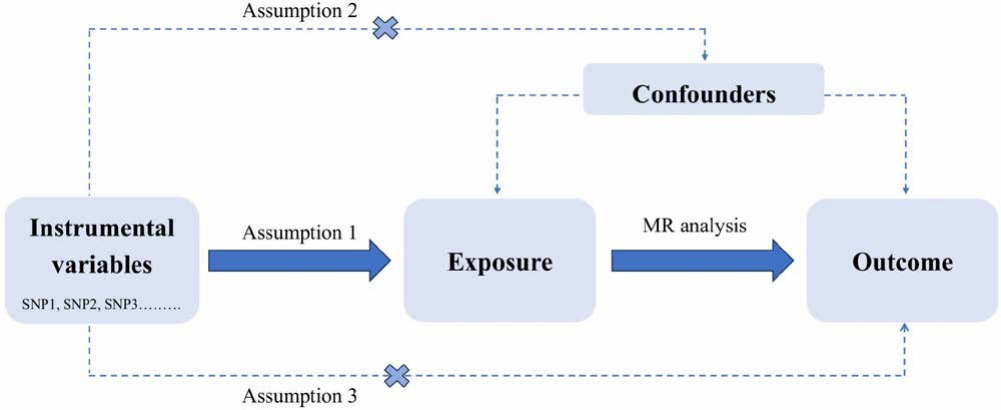
Flowchart of 3 assumptions.

A 2-sample bidirectional MR approach was employed to assess the causal relationships between anthropometric traits and BrS (Fig. 1). The study design was guided by the Strengthening the Reporting of Observational Studies in Epidemiology using Mendelian Randomization (STROBE-MR).^[17,18]^ The data utilized in our analysis were sourced from publicly available repositories. As this study utilized publicly available genome-wide association study (GWAS) summary statistics, additional ethical approval was not required. MR analysis relies on 3 core assumptions (Fig. 2): relevance: strong correlation between the instrumental SNPs and the exposure; independence: the instrumental variables remain unaffected by confounding influences; exclusion: the instrumental variables should affect the outcome variables exclusively through the exposure factors.

### 2.2. Exposures

The exposures in this study included key indicators related to birth weight, childhood obesity, adult obesity, fat distribution, and body composition. Measures of childhood obesity comprised childhood obesity status, childhood BMI, and comparative body size at age 10 (CBS-10). Indicators of adult obesity included adult BMI, as well as categorized overweight and obesity status. Fat distribution traits encompassed waist circumference, hip circumference, WHR, waist-to-hip ratio adjusted for body mass index (WHR_adj_BMI), percent liver fat, abdominal subcutaneous adipose tissue volume, and visceral adipose tissue volume. Body composition parameters included whole-body fat mass (WBFM), whole-body fat-free mass (WBFFM), WBWM, and BMR.

To reduce the risk of population stratification bias, only publicly available GWAS summary statistics from individuals of European ancestry were used. These data were divided into discovery and replication samples. The detailed data sources are listed in Table S1, Supplemental Digital Content, https://links.lww.com/MD/Q217.

#### 2.2.1. Data sources for birth weight and childhood obesity

Genetic associations for birth weight (n = 133,903/298,142 for discovery/replication),^[19,20]^ childhood obesity (n = 13,848/21,309),^[21,22]^ and childhood BMI (n = 356,668/39,620)^[23,24]^ were obtained from the Early Growth Genetics Consortium (http://egg-consortium.org/). Genetic instruments for CBS-10 were derived from UK Biobank participants (aged 40–69 years) who retrospectively reported their body size at age 10 compared to their peers. The discovery dataset included 453,169 individuals (n_female = 246,511; n_male = 206,658),^[25]^ with replication data comprising 454,718 individuals from the MRC-IEU UK Biobank GWAS pipeline.

#### 2.2.2. Data sources for adult obesity (general adiposity)

Genetic associations for adult BMI were obtained from GWAS summary statistics, comprising 236,781 individuals in the discovery dataset^[26]^ and 806,834 individuals in the replication dataset.^[27]^ Overweight and obesity classifications followed World Health Organization criteria based on adult BMI (kg/m²): overweight (25.0–29.9) and obesity (≥30), with subclassifications defined as class 1 obesity (30.0–34.9), class 2 (35.0–39.9), and class 3 (≥40).^[28]^

Discovery data for overweight status were obtained from the Genetic Investigation of Anthropometric Traits (GIANT) Consortium (n = 158,855),^[29]^ with replication data derived from a separate GWAS cohort (n = 429,567).^[30]^ Adult obesity datasets originated from Finnish population cohorts (discovery: n = 218,735; replication: n = 453,592). Obesity class-specific genetic association data (class 1 [n = 98,697], class 2 [n = 72,546], and class 3 [n = 50,364]) were obtained from the GIANT Consortium.^[29]^

#### 2.2.3. Data sources for fat distribution (abdominal/central adiposity)

Discovery data for waist circumference (n = 232,101) and hip circumference (n = 213,038) were obtained from the GIANT Consortium,^[31]^ with replication datasets from the MRC-IEU UK Biobank GWAS pipeline (n = 462,166 and 462,117, respectively). WHR data included 212,244 individuals in the discovery set^[31]^ and 697,734 individuals in the replication set.^[27]^ For WHR_adj_BMI, the discovery dataset comprised 458,349 individuals,^[32]^ while the replication dataset included 694,649 participants.^[27]^ GWAS data for percent liver fat, abdominal subcutaneous adipose tissue volume, and visceral adipose tissue volume were obtained from a cohort of 32,860 individuals.^[33]^

#### 2.2.4. Data sources for body composition

Discovery datasets for WBFM (n = 330,762), WBFFM (n = 331,291), WBWM (n = 331,315), and BMR (n = 331,307) were obtained from the Neale Lab (http://www.nealelab.is/uk-biobank) under ID ukb-a-265, ukb-a-266, ukb-a-267, ukb-a-268 within the UK Biobank, respectively. Replication data were sourced from the MRC-IEU UK Biobank GWAS pipeline and included WBFM (n = 454,137), WBFFM (n = 454,850), and WBWM (n = 454,888). Replication data for BMR included 534,045 individuals.^[34]^

### 2.3. Outcome

The outcome of interest, BrS, was analyzed using GWAS summary statistics from 12,821 individuals of European ancestry, including 2820 cases and 10,001 controls.^[35]^

### 2.4. Genetic instrument selection

Instrumental variables were selected based on the following criteria: genome-wide significance (*P* < 5 × 10^−8^, or *P* < 1 × 10^−5^ for exposures with fewer than 20 instruments); linkage disequilibrium clumping with *r*² < 0.001 and a window size > 10,000 kb^[36]^; and *F*-statistic > 10 to ensure instrument strength. Allele alignment was conducted between exposure and outcome datasets. When SNPs were unavailable, proxy variants were used. Ambiguous or palindromic SNPs, as well as those with a minor allele frequency < 0.01, were excluded from the analysis.

### 2.5. Statistical analysis

The primary analysis utilized inverse variance weighted (IVW) regression, while supplementary analyses employed MR-Egger and the weighted median approach. Comprehensive sensitivity analyses were conducted, including Cochran *Q* test, the MR-Egger intercept test, the MR-PRESSO global test, and leave-one-out analysis. Heterogeneity was assessed using Cochran *Q* test, while horizontal pleiotropy was evaluated via the MR-Egger intercept and MR-PRESSO global tests. In addition, we also employed the MR-PRESSO outlier-corrected test to detect potential outliers that could introduce horizontal pleiotropy and provide corrected MR estimates after removing these outliers. The stability of the results was assessed using leave-one-out analysis.

To account for multiple testing, a Bonferroni correction was applied to adjust *P*-values. A *P*-value <.0023 (0.05/22, where 22 denotes the number of exposure traits excluding CBS-10 gender stratification) was considered strong evidence of a causal association. Results with *P*-values between .0023 and .05 were regarded as suggestive associations. Reverse MR analysis followed the same protocols. All analyses were performed in R version 4.4.0 (R Foundation for Statistical Computing, Vienna, Austria) using the TwoSampleMR and MR-PRESSO packages.

## 3. Results

### 3.1. Genetic instrument information

The number of SNPs extracted from the relevant GWAS summary statistics to serve as genetic instruments ranged from 7 to 547 (Table S2, Supplemental Digital Content, https://links.lww.com/MD/Q217).

### 3.2. Associations between anthropometric traits and BrS risk

#### 3.2.1. Birth weight and childhood obesity

**Figure 3.**
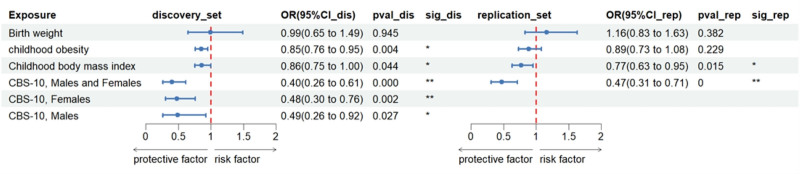
Associations of genetically predicted birth weight and childhood obesity with Brugada syndrome. ** Suggestive association (*P* < .0023), * significant association (*P* < .05 and >.0023). CI = confidence interval, OR = odds ratio.

MR analyses revealed that genetically predicted CBS-10 (per 1-SD increase; discovery set: odds ratio [OR]_IVW_ = 0.40, 95% confidence interval [CI]: 0.26–0.61, *P* = 2.07 × 10^−5^; replication set: OR_IVW_ = 0.47, 95% CI: 0.31–0.71, *P* = 3.68 × 10^−4^) was significantly inversely associated with BrS risk. Genetically predicted childhood obesity (discovery set: per log-odds ratio increase, OR_IVW_ = 0.85, 95% CI: 0.76–0.95, *P* = 3.55 × 10^−3^) and childhood BMI (discovery set: OR_IVW_ = 0.86, 95% CI: 0.75–1.00, *P* = .044; replication set: OR_IVW_ = 0.77, 95% CI: 0.63–0.95, *P* = .015) showed suggestive inverse associations with BrS risk. No significant association was observed between genetically predicted birth weight and BrS (*P* > .05; Fig. 3; Table S2, Supplemental Digital Content, https://links.lww.com/MD/Q217).

After Bonferroni correction, the associations for CBS-10 remained significant (Table S3, Supplemental Digital Content, https://links.lww.com/MD/Q217). Weighted median analyses further supported this association. Sensitivity analyses revealed significant heterogeneity (*P* < .05; Table S4, Supplemental Digital Content, https://links.lww.com/MD/Q217). MR-PRESSO identified outliers (global test *P* < .05), but the association persisted even after outlier removal (Table S6, Supplemental Digital Content, https://links.lww.com/MD/Q217).

Subgroup analyses demonstrated that genetically predicted female CBS-10 was significantly inversely associated with BrS risk (per 1-SD increase; OR_IVW_ = 0.48, 95% CI: 0.30–0.76, *P* = 1.65 × 10^−3^), while male CBS-10 showed a suggestive inverse association (per 1-SD increase; OR_IVW_ = 0.49, 95% CI: 0.26–0.92, *P* = .027). Sensitivity analyses consistently supported these findings. Heterogeneity was detected in female CBS-10 analyses (Cochran *Q* test *P* < .05), but the association remained robust after MR-PRESSO outlier correction (Table S6, Supplemental Digital Content, https://links.lww.com/MD/Q217).

#### 3.2.2. Adult obesity

**Figure 4.**
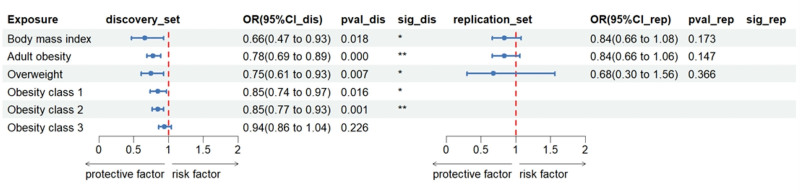
Associations of genetically predicted adult obesity with Brugada syndrome. ** Suggestive association (*P* < .0023), * significant association (*P* < .05 and >.0023). CI = confidence interval, OR = odds ratio.

Genetically predicted obesity was significantly inversely associated with BrS risk (discovery set: OR_IVW_ = 0.78, 95% CI: 0.69–0.89, *P* = 1.42 × 10^−4^). Suggestive inverse associations were observed for genetically predicted higher BMI (discovery set: OR_IVW_ = 0.66, 95% CI: 0.47–0.93, *P* = .018) and overweight status (discovery set: OR_IVW_ = 0.75, 95% CI: 0.61–0.93, *P* = .007). Subgroup analyses indicated that genetically predicted obesity class 2 was significantly inversely associated with BrS risk, while obesity class 1 showed an inverse association (Fig. 4).

After Bonferroni correction, the association between obesity and obesity class 2 remained significant (Table S3, Supplemental Digital Content, https://links.lww.com/MD/Q217). Weighted median analyses supported the association with obesity but not with obesity class 2. MR-PRESSO identified outliers in the obesity analyses (global test *P* < .05), but the association persisted after outlier correction (Table S6, Supplemental Digital Content, https://links.lww.com/MD/Q217).

#### 3.2.3. Fat distribution

**Figure 5.**
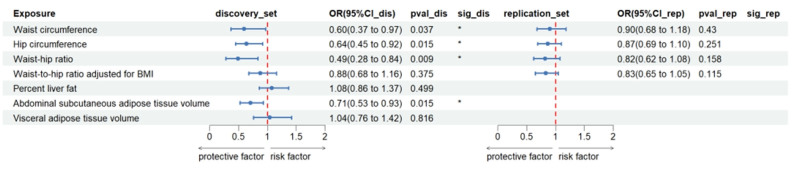
Associations of genetically predicted fat distribution with Brugada syndrome. ** Suggestive association (*P* < .0023), * significant association (*P* < .05 and >.0023). CI = confidence interval, OR = odds ratio.

In the discovery set, genetically predicted waist circumference (OR_IVW_ = 0.60, 95% CI: 0.37–0.97, *P* = .037), hip circumference (OR_IVW_ = 0.64, 95% CI: 0.45–0.92, *P* = .015), WHR (OR_IVW_ = 0.49, 95% CI: 0.28–0.84, *P* = .0093), and abdominal subcutaneous adipose tissue volume (OR_IVW_ = 0.71, 95% CI: 0.53–0.93, *P* = .014) exhibited suggestive inverse associations with BrS risk. No significant associations were observed for WHR_adj_BMI, percent liver fat, or visceral adipose tissue volume (*P* > .05; Fig. 5).

#### 3.2.4. Body composition

**Figure 6.**
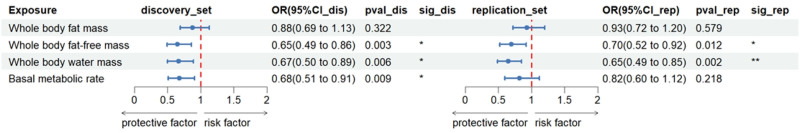
Associations of genetically predicted body composition with Brugada syndrome. ** Suggestive association (*P* < .0023), * significant association (*P* < .05 and >.0023). CI = confidence interval, OR = odds ratio.

Genetically predicted WBFFM (discovery set: OR_IVW_ = 0.65, 95% CI: 0.49–0.86, *P* = 3.12 × 10^−3^; replication set: OR_IVW_ = 0.70, 95% CI: 0.52–0.92, *P* = .012), WBWM (discovery set: OR_IVW_ = 0.67, 95% CI: 0.50–0.89, *P* = .006; replication set: OR_IVW_ = 0.65, 95% CI: 0.49–0.85, *P* = 2.09 × 10^−3^), and BMR (discovery set: OR_IVW_ = 0.68, 95% CI: 0.51–0.91, *P* = .009) were inversely associated with BrS risk. No association was observed for WBFM (*P* > .05; Fig. 6).

After Bonferroni correction, the association for WBWM (replication set) remained significant (Table S3, Supplemental Digital Content, https://links.lww.com/MD/Q217). Sensitivity analyses revealed heterogeneity (Cochran *Q* test, *P* < .05; Table S4, Supplemental Digital Content, https://links.lww.com/MD/Q217) and horizontal pleiotropy (Egger intercept, *P* < .05; Table S5, Supplemental Digital Content, https://links.lww.com/MD/Q217). MR-PRESSO identified outliers (global test, *P* < .05), but the association persisted after outlier correction (Table S6, Supplemental Digital Content, https://links.lww.com/MD/Q217).

### 3.3. Associations between BrS and anthropometric traits

**Figure 7.**
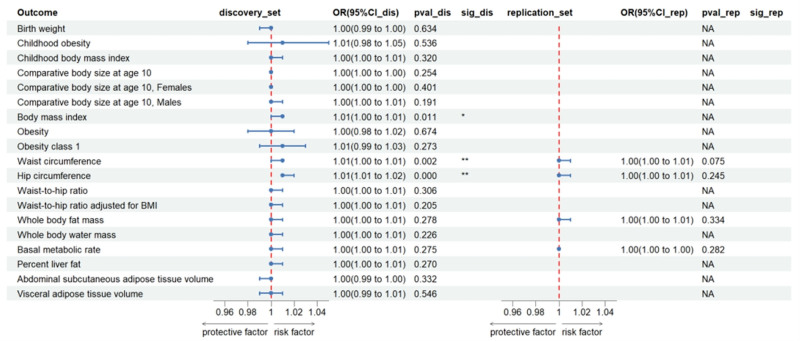
Associations of genetically predicted Brugada syndrome with multiple anthropometric characteristics. ** Suggestive association (*P* < .0023), * significant association (*P* < .05 and >.0023). CI = confidence interval, OR = odds ratio.

Reverse MR analyses indicated that genetically predicted BrS was associated with increased risks of waist circumference (OR_IVW_ = 1.009, 95% CI: 1.003–1.015, *P* = 1.63 × 10^−3^), hip circumference (OR_IVW_ = 1.012, 95% CI: 1.006–1.017, *P* = 2.33 × 10^−5^), and BMI (OR_IVW_ = 1.007, 95% CI: 1.002–1.013, *P* = .0112) in the discovery set (Fig. 7; Table S7, Supplemental Digital Content, https://links.lww.com/MD/Q217). After Bonferroni correction, the association between waist and hip circumferences remained significant.

## 4. Discussion

This MR study systematically evaluated the causal relationships between anthropometric characteristics at different life stages and BrS risk. We identified a significant inverse causal association between genetically predicted CBS-10 and BrS risk. Similarly, genetically predicted childhood obesity, higher childhood and adult BMI, adult obesity, WBFFM, WHR, and BMR were also causally associated with a reduced risk of BrS. Reverse MR analysis indicated that genetic liability to BrS may predispose individuals to larger waist and hip circumferences. In contrast, no significant association was observed for genetically predicted birth weight. To our knowledge, this is the first MR analysis to establish causal connections between life-course anthropometrics and BrS. These findings offer novel insights into BrS pathophysiology and provide potential evidence that could inform early risk stratification and intervention strategies.

### 4.1. Birth weight and childhood obesity

Although birth weight and childhood obesity exhibit genetic correlations with adult obesity,^[37,38]^ genetically predicted childhood obesity, CBS-10, and elevated childhood BMI demonstrated inverse associations with BrS risk, while birth weight showed no association. This suggests that childhood, rather than the fetal period, may represent a critical window during which body composition influences cardiac electrophysiological development.

The pathogenic mechanisms of BrS primarily involve functional abnormalities in Na⁺, Ca²⁺, and K⁺ ion channels. Over 500 BrS-associated pathogenic genetic variants have been identified, with approximately 30% located in the SCN5A gene.^[2,3]^ From a developmental biology perspective, the dynamic remodeling of childhood adipose tissue might influence the cardiac repolarization process by modulating gene expression in cardiomyocyte ion channels. Leptin secreted from prepubertal adipose tissue may regulate SCN5A mRNA expression via the SOCS3 and gp130-STAT3 pathways,^[39,40]^ potentially reducing BrS risk. Additionally, childhood obesity may enhance insulin sensitivity and secretion,^[41]^ subsequently altering myocardial electrophysiology through modulation of potassium channel currents.^[42]^ These mechanisms may explain the observed protective effect and warrant further investigation.

### 4.2. Adult obesity

BMI is a widely used metric for assessing general obesity, primarily reflecting overall body fat levels.^[43]^ MR analyses revealed inverse associations between genetically predicted increases in BMI and BrS risk. Subgroup analyses further demonstrated negative correlations for overweight (BMI 25–29.9 kg/m²), obesity (≥30 kg/m²), obesity class 1 (30–34.9 kg/m²), and class 2 (35–39.9 kg/m²), while no association was found for obesity class 3 (≥40 kg/m²).

This nonlinear pattern challenges the traditional assumption of linearity and supports the concept of an “obesity paradox.” Several mechanisms may underlie these findings: BMI-associated reductions in testosterone levels in males^[44]^; greater energy reserves in individuals with higher BMI, which may offer protection during physiological stress^[45,46]^; and reduced brown adipose tissue in overweight/obese individuals, potentially lowering the risk of febrile-triggered BrS.^[47]^ The lack of association in obesity class 3 may reflect impaired lipid storage capacity and subsequent visceral lipid overflow.^[48]^

### 4.3. Adipose distribution

Compared to BMI, which inadequately distinguishes regional fat distribution and fails to differentiate subcutaneous from visceral adiposity, WHR is a more accurate marker of abdominal obesity and better reflects visceral fat accumulation.^[43]^ Forward MR analyses revealed inverse associations between genetically predicted waist circumference, hip circumference, WHR, and abdominal subcutaneous adipose tissue volume with BrS risk, whereas no associations were observed for WHRadjBMI, percent liver fat, or visceral adipose tissue volume. Reverse MR analyses showed positive associations between BrS and both waist and hip circumferences.

The observed protective associations may be mediated by the metabolic buffering capacity of subcutaneous adipose tissue, which can expand to prevent ectopic fat deposition and associated metabolic dysfunction.^[49–52]^ Conversely, the reverse associations suggest that BrS may influence body composition through mechanisms such as: BrS-related exercise limitations promoting abdominal fat accumulation^[53]^; and *SCN10A* mutation-induced sympathetic imbalance affecting central fat distribution.^[54–58]^

### 4.4. Body composition

Fat mass and fat-free mass, as body composition indices, address BMI’s limitation in distinguishing between adipose and lean tissue. Fat mass quantifies adipose tissue, while fat-free mass encompasses muscles, bones, organs, ligaments, tendons, and water.^[59]^ BMR, which is positively correlated with feeding rate, is a major component of daily energy expenditure and is closely linked to fat-free mass.^[60,61]^ MR analyses showed that genetically predicted WBFFM, BMR, and WBWM were inversely associated with BrS risk, whereas fat mass showed no association. The protective effect of fat-free mass may be partly mediated by irisin, a myokine that improves myocardial Ca^2+^ handling.^[62]^ Accordingly, interventions such as resistance training and protein supplementation aimed at increasing fat-free mass could represent potential preventive strategies against BrS,^[63,64]^ though clinical validation is needed.

### 4.5. Strengths and limitations

The MR design minimized confounding and reverse causation, while the use of large sample sizes and multisource GWAS data improved statistical power and robustness. However, several limitations must be acknowledged: potential residual pleiotropy remains a concern, and further validation through multivariable MR is warranted.^[65]^ Despite extensive sensitivity analyses, we cannot entirely exclude the possible influence of pleiotropy or heterogeneity. Potential confounding factors (such as nutrition, physical activity, lifestyle, environmental conditions, and socioeconomic status) may also affect the outcomes. Specifically, these include maternal prepregnancy and gestational factors (e.g., weight and nutritional status) for birth weight; postnatal feeding practices, physical activity levels, insufficient sleep, screen time exposure, parental obesity, and unfavorable parental dietary and lifestyle habits for childhood obesity; and unhealthy diet, low physical activity, psychological stress or mental health disorders, poor sleep patterns, as well as comorbidities, medication use, and genetic factors for adult obesity.^[66,67]^ The limited sample size for BrS may reduce the ability to detect weak associations. The exclusive focus on individuals of European ancestry limits the generalizability of our findings, underscoring the necessity for validation in non-European populations.^[68]^

### 4.6. Future directions

Future research should develop SCN5A-mutant humanized mouse models to investigate the effects of adipose depot transplantation on cardiac electrophysiology. Body composition metrics should be incorporated into clinical risk prediction models using machine-learning approaches. In addition, single-cell spatial transcriptomics should be applied to map interactions within the adipose–cardiac microenvironment.

From a clinical perspective, BrS patients are advised to adopt personalized exercise regimens under professional supervision, considering individual BMI, physical fitness, comorbidities, and obesity-related conditions. To mitigate early risks, maintaining BMI within the upper-normal range and preserving fat-free mass through appropriate weight management, regular physical activity, and a balanced diet is recommended.

## 5. Conclusion

In conclusion, this study identified childhood obesity, adult obesity, and WBFFM as potential protective factors against BrS. These findings suggest that maintaining BMI within the upper-normal range, while increasing the fat-free mass proportion through resistance training and protein supplementation during childhood or adulthood, may represent effective strategies for mitigating BrS risk.

## Acknowledgments

The authors gratefully acknowledge the participants and investigators of all genome-wide association studies whose publicly available data were utilized in this research, including the Early Growth Genetics (EGG) Consortium, FINNGEN, UK Biobank, the GIANT Consortium, and the Neale Lab. We extend special thanks to the Brugada Syndrome GWAS consortium for providing outcome data. Finally, we acknowledge the developers of the TwoSampleMR and MR-PRESSO R packages for their open-source analytical tools.

## Author contributions

**Conceptualization:** Sina Liu, Hongxu Liu, Wenlong Xing.

**Data curation:** Sina Liu, Siqing Wang.

**Formal analysis:** Sina Liu, Mingxuan Li.

**Funding acquisition:** Sina Liu, Hongxu Liu.

**Methodology:** Juju Shang, Wenlong Xing, Wei Liu.

**Project administration:** Juju Shang, Shenglei Qiu, Xiaolei Lai.

**Software:** Sina Liu, Mingxuan Li, Siqing Wang.

**Supervision:** Shenglei Qiu, Xiaolei Lai.

**Validation:** Sina Liu, Mingxuan Li, Siqing Wang.

**Visualization:** Wei Liu.

**Writing – original draft:** Sina Liu, Mingxuan Li, Siqing Wang.

**Writing – review & editing:** Juju Shang, Hongxu Liu, Wenlong Xing, Wei Liu, Shenglei Qiu, Xiaolei Lai.

## Supplementary Material


